# Supply- and demand-side drivers of the change in the sugar density of food purchased between 2015 and 2018 in Great Britain

**DOI:** 10.1017/S0007114524001806

**Published:** 2025-03-28

**Authors:** Mathilde Gressier, Gary S. Frost, Zoe Hill, Danying Li, Jack Olney, Elisa Pineda, Victoria Targett, Michelle Young, Franco Sassi

**Affiliations:** 1 Section for Nutrition Research, Department of Metabolism, Digestion and Reproduction, Faculty of Medicine, Imperial College London, London, UK; 2 Centre for Health Economics & Policy Innovation, Department of Economics & Public Policy, Imperial College Business School, London, UK; 3 Office for Health Improvement & Disparities, UK Department of Health and Social Care, London, UK; 4 The George Institute for Global Health UK, School of Public Health, Imperial College London, London, UK

**Keywords:** Soft drink tax, Reformulation, Consumer behavior, Sugar purchase

## Abstract

The UK government launched a two-component sugar-reduction programme in 2016, one component is the taxation of sugar-sweetened beverages, the Soft Drinks Industry Levy, and the second is a voluntary sugar reduction programme for products contributing most to children’s sugar intakes. These policies provided incentives both for industry to change the products they sell and for people to change their food and beverage choices through a ‘signalling’ effect that has raised awareness of excess sugar intakes in the population. In this study, we aimed to identify the relative contributions of the supply- and demand-side drivers of changes in the sugar density of food and beverages purchased in Great Britain. While we found that both supply- and demand-side drivers contributed to decreasing the sugar density of beverage purchases (reformulation led to a 19 % reduction, product renewal 14 %, and consumer switching between products 8 %), for food products it was mostly supply-side drivers (reformulation and product renewal). Reformulation contributed consistently to a decrease in the sugar density of purchases across households, whereas changes in consumer choices were generally in the opposite direction, offsetting benefits of reformulation. We studied the social gradient of sugar density reduction for breakfast cereals, achieved mostly by reformulation, and found increased reductions in sugar purchased by households of lower socio-economic status. Conversely, there was no social gradient for soft drinks. We conclude that taxes and reformulation incentives are complementary and combining them in a programme to improve the nutritional quality of foods increases the probability of improvements in diet quality.

In 2016, the UK government launched the first chapter of its plan and commitments to reduce childhood obesity^(1)^. Two actions focused on reducing sugar intakes – the Soft Drinks Industry Levy (SDIL) and the voluntary sugar reduction programme^(2)^.

The SDIL is a tax on sugar-sweetened beverages, which was enforced in the UK in April 2018. The tax rate increases with sugar density – drinks with less than 5 g of sugar per 100 ml are exempted, those with more than 8 g of sugar per 100 ml are subject to a higher tax rate of 24 pence/l and drinks between the two thresholds are taxed at a lower rate of 18 pence/l. The SDIL was designed in this way to deliver its stated primary objective of incentivising sugar-sweetened beverage manufacturers and importers to reduce the sugar content of the beverages they produce and shift their sales to lower-sugar products. In addition, like all sugar-sweetened beverages taxes, it was anticipated that the SDIL would increase the price of some taxed beverages, with expected additional benefits in terms of reduced consumer demand for higher sugar products.

The sugar reduction programme was designed to promote voluntary reformulation by businesses across all sectors of the food industry – supermarkets, manufacturers, cafes, pubs, restaurants, quick service restaurants, takeaway and delivery – to reduce by 20 % by 2020 the levels of sugar in products that contributed the most to children’s sugar intakes. The product categories covered by the programme include breakfast cereals, yogurts, cakes, biscuits, puddings, ice cream and sweet and chocolate confectionery. Guidelines for businesses were published in March 2017. The programme builds on the success of, and follows a similar model to, the voluntary salt reduction programme, which has been running since 2006.

The fourth sugar reduction progress report showed that the average sugar density of retailer and manufacturer branded beverages purchased in Great Britain and subject to the SDIL decreased by 46 % between 2015 and 2020^(3)^. A 43 % reduction was seen for the out of home sector. The report also showed that, for retailer and manufacturer branded products, all food categories included in the voluntary programme had reduced sugar levels between 2015 and 2020, although this was marginal for chocolate confectionary. For example, the sales-weighted average total sugar per 100 g of breakfast cereals, and of yoghurts and fromage frais, decreased by 14·9 % and 13·5 %, respectively. The picture was more mixed for the out-of-home sector. However, no food category across either sector met the 20 % sugar reduction ambition.

Reformulation of high-sugar foods would be expected to translate into a reduction in sugar intake. Data from the National Diet and Nutrition Survey (2019) shows that sugar intakes have fallen for some age groups which, in older children and adolescents, appears to be partly driven by soft drinks contributing less to sugar intakes, likely because of the changes made to drinks included in the SDIL^(3)^. Rogers *et al.* (2023) estimate that the UK SDIL was associated with an 8 % relative reduction in obesity levels in girls aged 10/11 years, equivalent to prevention of 5234 cases of obesity per year in that group, with the greatest reductions in the most deprived areas^(4)^.

However, reductions in sugar intake can be hindered by changes in consumer behaviour, such as increases in the overall quantity of products consumed, or a shift to products with more sugar^(5)^. The overall reduction in average sugar levels, for the sugar reduction programme as a whole, is 3·5 %, substantially below the reductions achieved in some categories. This is because of large increases in sales of higher sugar products, such as sweet spreads and sauces (up 32 %) and chocolate confectionery (up 27·8 %), Conversely, the impact of reformulation on sugar intakes could be magnified if consumers did not change their food choices away from those that had been reformulated or switched to lower-sugar products.

Consumer choices are driven by a range of factors, including changes in the wider food environment, government campaigns and health messaging and commercial advertising^(6)^. The SDIL and sugar reduction programme created different incentives for change in different types of products. For sugar sweetened beverages, the tax incentivised both reformulation and shifts in consumer choices towards the manufacture and purchase of lower sugar products as this was a mandated measure that created a financial incentive for change. The voluntary programme relied on businesses taking action to reduce sugar levels as part of their regular review and reformulation of products; and the incentive of progress being monitored by both business and brand. The 2015 Public Health England (PHE) report *Sugar Reduction: The evidence for action* ^(2)^ recommended a wider range of evidence-based actions that could have supported the Sugar Reduction programme, but were not implemented by the Government.

The most recent evaluation of the voluntary sugar reduction programme showed that the sugar volumes purchased by British consumers dropped for beverages and for some foods^(7)^, but it did not attempt to identify the drivers of that outcome, that is, whether those changes were due to, for example, reformulation or changes in sales of products within the category towards those with lower sugar levels^(7,8)^.

To understand which factors drove the reduction in sugar density in both beverage and food products, we carried out a decomposition analysis to estimate the relative contributions of supply- and demand-side drivers, taking into consideration the different levels of consumer awareness generated by the policies put in place for different product categories.

There is established evidence of social disparities in diets in the UK. Data show that households of a higher socio-economic status, measured by income or occupation, purchase smaller proportions of less healthy foods and purchase and consume smaller amounts of sugar than households of lower socio-economic status^(9–11)^. In addition, the detrimental health outcomes associated with poor diet, alongside other health-related behaviours, are exacerbated among people with higher levels of deprivation^(12)^. This data demonstrate that an assessment of the drivers leading to changes in sugar intake should consider socio-economic differences wherever possible. This study investigated whether changes in the sugar density of purchases of drinks and foods differed across households of different social status. Specifically, we investigated whether the SDIL and sugar reduction programme triggered different changes in food choices in different socio-economic groups.

## Methods

### Methods and materials

The study was undertaken in two parts. First, a population-level analysis was carried out to evaluate the drivers of change in the sugar density of purchased products, on average across households in Great Britain (GB). Next, a household-level analysis was carried out to explore how the above drivers differed between households.

### Data: surveys of household purchases for at-home consumption

Purchase data were used in this study and are taken as a proxy for consumption. Consumption data are available in the UK, typically from surveys like the National Diet and Nutrition Survey. However, we have preferred to use purchase data for this study for the following two reasons: (a) purchase data include nutrient composition information at the individual product (brand) level, while consumption surveys typically include nutrient information imputed at a more aggregate level and (b) purchase data are available for a panel of households followed over several years. Purchase data, while not a direct measure of consumption, have been shown to be valuable in the evaluation of food and nutrition policies^(13)^.

#### Population-Level analysis: data used

The first study used data for the average sugar density of all food and drink purchased for at-home consumption across GB households, which was calculated using aggregated Kantar data for 2015 and 2019^(14)^.

The food and drink purchases made by GB households were taken from the dataset for Kantar Fast-Moving Consumer Goods take-home consumer panel. Approximately 30 000 households take part in the panel each year, with the same households taking part in different years (with a retention rate of about 85 %). The Kantar panel is built to be representative of the GB population, and survey weights were provided and used to build indicators representative of the population. Household purchases were self-reported using scanning equipment and shop receipts. All purchases of food and beverages that were brought into the home were recorded by the households taking part in the panel. The nutrition information for all products purchased is taken from food labels and updated regularly by Kantar. Hence, each individual branded product is linked to its nutrition information.

Aggregated data reflecting total annual sales of each product were used to estimate changes in sugar density for the whole GB population and their drivers (decomposition analysis). The average sugar density of purchases was obtained by weighting the sugar density of each product by the respective sales volume. Years used in the population-level analysis were 2015 (52 weeks ending 31 January 2016) and 2019 (52 weeks ending 8 September 2019).

Kantar data identify products using their barcode. The method used in this study, the decomposition method, needed to distinguish between products that stayed on the market (and were potentially reformulated) from products that exited or entered the market. To do that, products present across different years were matched (e.g. a supermarket own brand 500 g pack of cornflakes from the 2015 dataset was matched to the same product in the 2019 dataset). As the aim was to evaluate the impact of changes in products *vs*. changes in consumption patterns, a shift by consumers between two pack sizes of the same product was not considered as a change in shopping behaviour, but a change in the quantity chosen of the same product. Hence, product codes which had the same product description, belonged to the same category, and had the same manufacturer and brand, were grouped together as the same product. For example, all chocolate-covered digestive biscuits from the same brand were grouped as one product despite being sold in different pack sizes (for drinks, canned and bottle versions of the same product were kept separate for colas as canned colas and bottled colas, lemonades and soft drinks of other flavours are classified in different sub-categories in Kantar dataset). Then, the grouped products were then matched across the different years.

Kantar also collects data on the characteristics of each household included in the panel. These include household composition (age and gender of each panel member), income, socio-economic status, if the household is placed in a rural or urban area, the ethnicity of the main shopper and the life stage of the household. Definitions of socio-economic status from the UK Office for National Statistics were used, which classifies households into five categories, based on the occupation of the household reference person, from AB (higher and intermediate managerial, administrative and professional occupations) to E (unemployed and lowest grade occupations).

#### Household-Level analysis: data used

In the second part of the study, household-level data were used to estimate changes in sugar density over time for each household. The data source for this analysis was the same as for the population-level analysis (Kantar household panel survey), but this time disaggregated data were used. Survey weights were not used in the second part of the study as it compared changes in within-household purchases over time. The most recent household-level data from Kantar available to the authors at the time of analysis (from the years 2015 and 2018) were used.

The aim of this analysis was to obtain a better understanding of demographic drivers of changes in the sugar density of foods purchased for consumption at home. This analysis was carried out on two product categories: soft drinks and breakfast cereals. The scope of this analysis was more limited than that of the population-wide analysis because of the high access costs of disaggregated data and because of the resource intensity of this type of analysis. The two categories were chosen because they were found to have had the largest reductions in sugar density in the first years of the voluntary sugar reduction programme and following the announcement and implementation of the SDIL^(7)^. To understand if the levy resulted in changes in the consumption of drinks not in scope (e.g. pure fruit juices and water), the purchase of all types of non-alcoholic drinks was included in the analysis category ‘all drinks’.

For this analysis, purchases (volume and sugar from soft drinks) per adult equivalent were calculated for each household. This method, allowing the comparison of purchases from households of different sizes, is commonly used in similar studies^(15–17)^. The number of adult equivalents for each household was calculated by summing the estimated average requirement for energy of all households members (obtained from their gender and age) and dividing it by 2605 kcal, the estimated average requirement for energy for adult males^(18)^. For example, the number of adult equivalent of a family consisting of an adult men, an adult women and a girl aged 8 years is (2605 + 2079 + 1625)/2605 = 2·4. Then, volumes and sugar from all household purchases were divided by the number of adult equivalents to obtain purchases per adult equivalent.

#### Food categories and identification of food and drink included in the sugar reduction programme or soft drinks industry levy

Both analyses required the identification of drinks in the scope of the SDIL or foods included in the voluntary sugar reduction programme (categories detailed in PHE, 2017^(19)^).

Each food present in the Kantar dataset was classified into either one of the product categories included in the sugar reduction programme or to a separate list of excluded products for those foods not included in these categories. The classification was completed by following the technical guidance issued by PHE on what is included in, or excluded from, the categories in the sugar reduction products^(19)^ as well as using product categorisation completed by Kantar and the product description in the dataset. Similarly, drinks were classified as either being in or out of the scope of the SDIL. Drinks with a sugar content of less than 5 g/100 ml were classified as being within scope of the SDIL as this enabled an assessment of any shifts in purchasing towards lower sugar drinks. This is the same method that has been used by PHE and Office of Health Improvement and Disparities when evaluating sugar reductions delivered in products within the scope of the SDIL^(7)^. This classification was then checked with experts from PHE.

### Method for decomposing changes in sugar density

We used a decomposition method to assess changes in the sugar density of household purchases driven, respectively, by supply-side and demand-side factors. The method was first used in a food context by Griffith *et al.* to evaluate the drivers of the decrease in salt content of food purchases in the UK^(20)^. The decomposition analysis aimed to explain the respective roles of supply-side drivers (sugar reformulation of food products and product renewal: when food manufacturers remove some products from the market and/or introduce new ones) and demand-side drivers (changes in the types of products chosen by consumers, within and between categories, and in the quantities purchased) in the reductions seen over time in quantities of sugar purchased. The decomposition applies to a component density, in this study it is sugar density, defined as per equation (1):
(1)

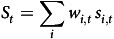

with 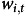 the proportion of product 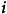 purchased defined as 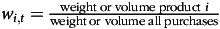 and 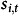 the sugar content per 100 ml or 100 g of the product 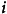 purchased. When food and drinks were considered together (e.g. per 100 g of products in the average purchase basket), it was hypothesised that 1 ml of drink equals 1 g in weight. Where a drink needs to be diluted before consumption (e.g. fruit squash) the analysis used the sugar content for the, for example, squash once it was in its ready-to-drink or diluted form (according to the instructions given on pack). The decomposition algorithm decomposes the change in sugar density into three components:

- A change from reformulation: a change in the sugar density of a product, keeping its volumes purchased constant.
- A change from switching between products: the change in volume purchased for products in the market between the two time points and where the product(s) were not reformulated.
- A change from product renewal: the change in volume purchased for products that only appear in the dataset at one time point, that is, products that were available in 2015 but are not included in the second (2018) dataset, or new products only included in the second (2018) dataset.

The details of, and equation for, the decomposition method are explained in another paper^(21)^

The decomposition method was used in both analyses:

- Population-level analysis: decomposition of the sugar density of all purchases for at-home consumption of the GB population (method applied to all purchases, foods included in the sugar reduction programme, soft drinks and by food categories of the sugar reduction programme).
- Household-level analysis: decomposition of the sugar density of each household’s purchases of all drinks, soft drinks and breakfast cereals purchased for at-home consumption.

### Explaining heterogeneity in household-level data (second study)

The household-level decomposition enabled exploration of the heterogeneity in changes in sugar density of purchases observed between households. After applying the decomposition equation for each household, we used linear regressions to investigate if the three effects (i.e. reformulation, product switching and product renewal) differed by demographic characteristics. For each category, each of the three effects was regressed against the baseline volume per adult equivalent purchased from this category, the baseline sugar density of purchases as a proxy for sugar preference and the demographic characteristics of the household, as in equation (2) below (the example is provided for the reformulation effect).
(2)

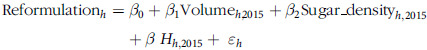





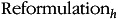
 is the reformulation effect calculated for household *h* obtained from the decomposition algorithm. 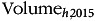 is the baseline volume of products purchased in 2015 by the household. This variable is an indicator differentiating high consumers of the product category studied, from low. 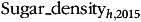 is the average sugar density of purchases by the household in 2015. This variable controls for the preference for sugary products at baseline. 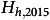 is a vector of socio-demographic characteristics of the households in 2015, including household size, income, social grade, if the household is placed in a rural or urban area, the ethnicity of the main shopper and the life stage of the household.

Lastly, the change in the quantity of sugar purchased per adult equivalent in a household was regressed against the result of the decomposition analysis (i.e. reformulation, product switching and product renewal) and against household characteristics to test the predictive power of each decomposition effect on the change in sugar purchases, as in (3) below. This regression was done for the three product categories separately.
(3)

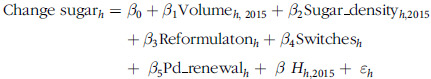





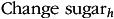
 is the difference in the quantity (in grams/year) of sugar purchased by the household per adult equivalent between 2015 and 2018.

## Results

### Population-Level analysis: descriptive statistics

The population-level analysis covered 149 060 products, of which 41 340 were present both in 2015 and in 2019 (Table 1). Twenty-one percent of the products in the database were within the scope of the SDIL or sugar reduction programme.

**Table 1.** Number of grouped products by category and share of each category in the average basked purchased by GB households in 2015 and 2019

	Number of products			Share of the average basket (in volume)	
	Products in 2015 only	Products in 2015 and 2019 (continued products)	Products in 2019 only (new products)	in 2015	in 2019
*Products not in SDIL or SRP*	43 387	32 222	41 710	79 %	79 %
Soft drinks	2002	1638	1671	11 %	12 %
Yoghurts	710	443	911	1·6 %	1·5 %
Biscuits	1755	1224	1776	1·5 %	1·5 %
Breakfast cereal	538	695	581	1·5 %	1·4 %
Puddings	1498	1042	1448	1·2 %	1·1 %
Ice cream	859	527	870	1·0 %	1·0 %
Chocolate confectionery	1103	1464	1903	0·9 %	1·0 %
Cakes	1214	519	472	0·9 %	0·5 %
Morning goods	453	258	209	0·9 %	0·8 %
Sweet confectionery	1124	1113	1224	0·4 %	0·5 %
Spreads and sauces	137	195	165	0·1 %	0·1 %
TOTAL	54 780	41 340	52 940	100 %	100 %

GB, Great Britain; SDIL, Soft Drinks Industry Levy; SRP, Sugar Reduction Programme.

Data source: aggregated Kantar panel.

### Changes in the sugar density of purchases between 2015 and 2019

**Fig. 1.**
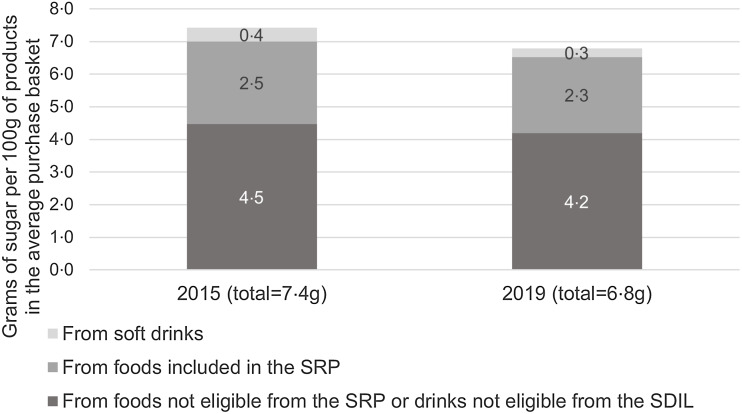
Average sugar density of food and drinks purchased for at-home consumption, in 2015 and 2019. SRP, Sugar Reduction Programme; SDIL, Soft Drinks Industry Levy. Data source: aggregated Kantar panel.

The average sugar density of purchased products decreased from 7·4 g/100 g in 2015 to 6·8 g in 2019 (Fig. 1). This decrease in sugar density was visible in foods included in the sugar reduction programme (SRP) (from 2·5 to 2·3 g), soft drinks (from 0·4 to 0·3 g) but also foods and drinks not included in either the sugar reduction programme or the SDIL (4·5–4·2 g).

### Decomposition of purchases for at-home consumption

For foods included in the sugar reduction programme, the sugar density decreased by 2 %, with most of this reduction coming from reformulation (Table 2). The average sugar density of drinks (all soft drinks) purchased by the population decreased by 41 %, with this decrease being explained by all three effects (Table 2) although the effects of reformulation and product renewal were larger than the product switching effect.

**Table 2.** Average sugar density of food and drink products purchased for at-home consumption in 2015 and 2019 and results of the decomposition of the change in the average sugar density into effects of reformulation, product switching and product renewal

Average sugar densities in g/100 mg or g/100 ml	Soft drinks	Foods in the SRP
Sugar density, 2015	3·7	25·4
Reformulation effect	–0·7	–0·6
Switching effect	–0·3	0·1
Product renewal effect	–0·5	–0·1
Sugar density, 2019	2·2	24·8
Change in sugar density (%)	–41 %	–2 %

‘All purchases’ means all food and drink items purchased in the Kantar panel; ‘Foods in the SRP’ means (solid) foods included in the Sugar Reduction Programme (SRP).

Data source: aggregated Kantar panel. The three effects (reformulation, product switching and product renewal) were obtained through the decomposition algorithm, applied at the population level to the sugar density of the average basket of food products purchased and of the average basket of drinks purchased. The sum of the three decomposition effects equals the difference in the sugar density.

The decomposition was applied to changes in the sugar density of all purchases in order to understand the extent to which foods and drinks included in the SRP and SDIL contributed to the decrease in sugar density observed for all purchases (Table 3). Despite having a lower sugar density at baseline (3·7 g/100 ml of drinks, Table 2), soft drinks contributed to the decrease in sugar density of the average basket to a similar degree to foods included in the SRP between 2015 and 2019 (–0·15 g and –0·20 g, respectively – Table 3). The reformulation effect was of a similar size for included foods and drinks, accounting, respectively, for a reduction of –0·06 g and –0·08 g of sugar per 100 g in the average basket of products purchased for at-home consumption (Table 3). This is the result of the combination of substantial reformulation of soft drinks (–19 % sugar density, Table 4) and a higher sugar density at baseline for included foods (25·4 g sugar /100 g, Table 2) but a lower level of reformulation (–2 % sugar density).

**Table 3.** Sugar content per 100 g of the average basket of products purchased for at-home consumption, for products included and not included in the programme

	Average basket in 2015	Reformulation effect	Switching effect	Product renewal effect	Average basket in 2019
From foods and drinks not included the SRP and SDIL	4·47 g	0·04 g (–0·6 %)	–0·06 g (–0·8 %)	–0·18 g (–2·4 %)	4·20 g
From foods included in the SRP	2·53 g	–0·06 g (–0·8 %)	–0·08 g (–1·1 %)	0·01 g (–0·2 %)	2·33 g
From soft drinks	0·42 g	–0·08 g (–1·0 %)	0·05 g (–0·7 %)	–0·07 g (–1 %)	0·26 g
Total average 100 g basket	7·42	–0·18	–0·19	–0·26	6·78

Data source: aggregated Kantar panel. The three effects (reformulation, product switching and product renewal) were obtained through the decomposition algorithm, applied at the population level to the sugar density of the average 100 g of food and drinks in the average purchase basket.

SDIL: soft drinks industry levy; SRP, sugar reduction programme.

**Table 4.** Decomposition of the change in the sugar density of the different categories included in the sugar reduction programme, 2015–2019, for foods purchased for at-home consumption

	Sugar density 2015 (g/100 g or g/100 ml)	Reformulation effect	Switching effect	Product renewal effect	Sugar density 2019 (g/100 g or g/100 ml)	% change in sugar density^a^
Soft drinks	3·7	–19 %	–9 %	–13 %	2·2	–41 %
Breakfast cereal	16·8	–10 %	–1 %	–3 %	14·3	–15 %
Yoghurts	12·4	–4 %	1 %	–10 %	10·7	–13 %
Cakes	34·3	–3 %	–2 %	–2 %	31·9	–7 %
Ice cream	18·7	–7 %	3 %	–2 %	17·5	–6 %
*Products not in SDIL or SRP*	*5·7*	–1 %	–2 %	–3 %	*5·3*	*–6 %*
Spreads and sauces	28·8	1 %	3 %	–10 %	28·1	–2 %
Puddings	18·9	–3 %	3 %	–1 %	18·6	–1 %
Biscuits	31·2	–1 %	–1 %	1 %	30·9	–1 %
Sweet confectionery	60·4	–1 %	0 %	0 %	60·4	0 %
Chocolate confectionery	53·8	1 %	0 %	0 %	53·9	0 %
Morning goods	12·5	1 %	–5 %	4 %	12·6	1 %

The sugar densities are in g/100 g or g/100 ml, and the three effects are % change compared with baseline sugar density. The sum of the three effects equals the total % change in the sugar density of the category. Data source: aggregated Kantar panel. The three effects (reformulation, switching and product renewal) were obtained through the decomposition algorithm, applied at the population level to the average sugar density of each food category.

SRP, sugar reduction programme.

a The % change in sugar density between 2015 and 2019 here are different than the same metric in the third year progress by industry report^(7)^ due to small differences in methodology (such as the classification of foods within the programme categories, or the baseline year for morning goods and cakes).

The individual food categories included in the sugar reduction programme experienced different changes in sugar density. For most categories, except for morning goods and chocolate or sweet confectionery, the sugar density decreased (Table 4). Breakfast cereals and yoghurts were the categories with the highest sugar reductions (–15 % and –13 %, respectively). The sugar reduction of these two categories came from different supply-side drivers: reformulation for breakfast cereals, and product renewal for yoghurts. Overall, the effects of reformulation and product renewal generally indicated a reduction in sugar density, while the effect from product switching was small and often resulted in an increase in sugar density (Table 4).

### Household-Level decomposition analysis

#### Unadjusted mean changes

On average, the sugar density of household purchases of all drinks, soft drinks and breakfast cereals (i.e. of the categories included in the household-level analysis) decreased significantly (Table 5). Decreases were of a magnitude of –1·5 g/100 g for breakfast cereals and –1·1 g and –1·3 g/100 ml for all drinks and soft drinks, respectively (Table 5, all three with a *P* value less than 0·00005). Nonetheless, the standard deviation of the change in sugar density was high (and higher than the mean) showing differences in change in sugar density across households.

**Table 5.** Mean volume and sugar purchased in a year and sugar density of purchases of all drinks, soft drinks and breakfast cereals by households purchasing these products, in 2015 and 2018

	Unit	All drinks^a^		Soft drinks		Breakfast cereals	
		Mean	sd	Mean	sd	Mean	sd
N households		18 512		18 343		18 355	
Volume in 2015	(kg/year or L/year)	175·9	186·7	92·9	127·5	17·3	16·9
Volume in 2018	(kg/year or L/year)	176·4	191·2	88·1·	122·5	16·6	16·3
Mean change in volume for the household	(kg/year or L/year)	0·59	125·9	–4·83^*^	86·3	–0·74^*^	11·5
		(*P* = 0·536)		(*P* < 0·001)		(*P* < 0·001)	
Mean change in volume per adult equivalent	(kg/year or L/year)	1·94^*^	71·1	–1·32^*^	49·1	–0·28^*^	6·7
		(*P* = 0·0022)		(*P* = 0·003)		(*P* < 0·001)	
Sugar density of purchases in 2015	(g/100 g or g/100 ml)	4·49	3·13	4·56	3·96	15·94	7·73
Sugar density of purchases in 2018	(g/100 g or g/100 ml)	3·44	2·69	3·27	2·98	14·43	7·46
Mean change in density	(g/100 g or g/100 ml)	–1·06^*^	2·64	–1·29^*^	3·58	–1·51^*^	7·68
		(*P* < 0·001)		(*P* < 0·001)		(*P* < 0·001)	
Sugar quantity purchased in 2015	(g/year)	6948	9082	3542	6653	2886	3337
Sugar quantity purchased in 2018	(g/year)	5087	6742	2272	4585	2505	2965
Mean change in sugar for the household	(g/year)	–1861^*^	6314	–1270^*^	4867	–381^*^	2401
		(*P* < 0·001)		(*P* < 0·001)		(*P* < 0·001)	
Mean change in sugar per adult equivalent	(g/year)	–886^*^	3315	–586^*^	2503	–185^*^	1345
		(*P* < 0·00005)		(*P* < 0·00005)		(*P* < 0·00005)	

Cells shows weighted means and (standard deviations), except for the number of households that is not weighted by the Kantar survey weights to ensure representativity of English households.

a “All drinks” are all non-alcoholic beverages purchased by households, including water and pure fruit juices.

* Means a significant paired *t*-test for household changes and adult-equivalent change in sugar, density and volume.

Data source: Kantar panel, household level.

In the sample of households considered in this study, with the product definitions and metrics used, the volume of all drinks (including water) purchased by households increased both per adult equivalent and for the average household, although not significantly, while the volume of soft drinks purchased decreased (Table 5). The volume of breakfast cereals purchased also decreased.

As a result of the decreased volume and sugar density of purchases, the mean quantity of sugar purchased per year per household, and per adult equivalent, decreased for the three categories between 2015 and 2018 (Table 5). Decreases were –886 g (sd 3315 g) per adult equivalent from all drinks, including –586 g (sd 2503 g) from soft drinks and –185 g (sd 1345 g) from breakfast cereals. The high standard deviations indicate that the spread of the difference in sugar purchased per household in 2015 and 2018 was wide, with some households deviating substantially from the average.

#### Heterogeneity of effects across households

Overall, the decomposition of the changes in sugar density for the three product categories showed similar patterns (online Supplementary Figs. 1–3). Supply-side driver effects (reformulation and product renewal effects) were relatively consistent and contributed to a decrease in sugar density of purchases in about 75 % of households (online Supplementary Figs. 1–3 and online Supplementary Table 1). On the other hand, the effect of households switching between products showed a larger variability, with about half of the households switching towards products with higher sugar levels and half switching towards products with lower sugar levels.

For all drinks and soft drinks, the means of the three effects are negative, that is, on average, reformulation, product renewal and product switching contributed to a decrease in the sugar density of purchases (online Supplementary Figs. 1 and 2 and online Supplementary Table 1). For soft drinks, the mean reformulation effect was twice the size of the product switching effect and three times the size of the product renewal effect. For breakfast cereals, the mean reformulation and product renewal effects were negative, while the mean product switching effect was null.

Linear regressions were performed to investigate how household characteristics were linked to the drivers of changes in sugar density of purchases. The regressions showed that the baseline volume of products purchased, preference for sugar, and demographic characteristics can partially predict the effects of reformulation, product switching, and product renewal (results in online Supplementary Tables 2–5). More importantly, the models explained 16 %, 20 % and 20 % of the variance in the switching effects of all drinks, soft drinks and breakfast cereals, respectively.

For all drinks and for soft drinks, the baseline sugar density of the purchases of a household was the main predictor of the switching effect. Households purchasing drinks of a higher sugar density in 2015 were more likely to switch to products with less sugar (*P* < 0·01). Households in which the main shopper was not white were more likely to switch to products containing more sugar than households with a white main shopper (*P* < 0·01). In addition, some household demographics predicted the switching effect. Only for all drinks, people of socio-economic status C2, D or E were more likely to switch to drinks with less sugar than households of socio-economic status A (all *P* < 0·01) (online Supplementary Table 2).

For breakfast cereals, the main predictors of the switching effect were the life stage of the household and the sugar density of purchases at baseline (online Supplementary Table 4). Households with children had a smaller decrease (or higher increase) in the sugar density of their purchases than households without children (online Supplementary Fig. 4). Households preferring breakfast cereals with a higher sugar density at baseline were more likely to switch to lower sugar products than households preferring lower-sugar-density products at baseline (online Supplementary Fig. 4).

### Role of supply- and demand-side drivers and demographics in explaining changes in sugar purchases

The switching effect was highly correlated with changes in the sugar density of purchases (Table 6). The correlation was weaker for reformulation and product renewal effects in the three categories.

**Table 6.** Correlations between the three effects and the change in sugar density of purchases by a household

Correlation with change in sugar density	All drinks	Soft drinks	Breakfast cereals
Reformulation effect	36 %	45 %	23 %
Switching effect	87 %	81 %	86 %
Product renewal effect	48 %	42 %	41 %

Data source: Kantar panel, household level.

Regression analyses showed that all drivers positively and significantly influenced the change in sugar purchased, in the three categories (online Supplementary Table 5). The driver of the largest change in sugar density was different for each category: product renewal for all drinks, product switching for soft drinks and reformulation for breakfast cereals.

In addition, households with a higher sugar density at baseline had a larger reduction in purchased sugar quantity than households with lower sugar density at baseline. The change in sugar purchased from the three categories was negatively correlated with the baseline volume of products purchased (online Supplementary Table 5).

The regression analyses included controls for demographic characteristics, some of which had an influence on the change in sugar purchased. Larger households were more likely to decrease their purchase of sugar from the three categories than smaller households (online Supplementary Table 5). There was a small gradient for the change in sugar purchased from soft drinks by socio-economic group (online Supplementary Fig. 5). Households of socio-economic group D purchased significantly less sugar from soft drinks than group A or B (the highest classes). On the other hand, households from group E (the lowest class) had a smaller decrease in their purchases than households from other socio-economic groups (online Supplementary Fig. 5).

There was a stronger gradient by socio-economic group in the change in sugar purchased from breakfast cereals, with consistent reductions of an increasing magnitude from groups A to E (online Supplementary Fig. 6). It should be noted that group E is quite different to other socio-economic groups in terms of the number of people in each household and the age of the main shopper.

## Discussion

This study shows that reductions in the sugar density of drinks purchased by households were driven by factors on both the supply side (reformulation and product renewal) and the demand side (consumer switches), while reductions in the sugar density of foods were mostly driven by supply-side factors.

### The roles of supply- and demand-side drivers

For products in the SRP, reformulation drove a reduction in sugar density in almost all categories investigated. For sweet spreads and sauces and morning goods, the reduction in sugar density was not driven by reformulation, but instead by-product renewal and the switching effect, respectively. For both drink categories (all drinks and soft drinks) and for breakfast cereals, supply-side drivers were, on average and for at least 75 % of households, contributing to decreases in the sugar density of purchases. This means that, between 2015 and 2018, manufacturers (including retailers, for own-brand products) chose to reduce the sugar density of products in the categories examined in this study, through reformulation and product renewal. Manufacturers reduced the sugar content of their products, delisted products (removed from sale) with higher sugar content and introduced new products with lower sugar content. Analysis at the population and household levels show that the average switching effect on the sugar density of purchased foods (for which only voluntary sugar reformulation targets applied) was negligible. However, on average, the switching effect for drinks contributed to lowering the sugar density of drinks, demonstrating that the SDIL was able to incentivise households to switch to drinks with lower sugar content. Given that our household-level analysis ends in 2018, it is legitimate to assume that this shift happened even before most changes in the prices of taxed drinks were made. The importance of supply-side drivers compared with demand-side ones has been found in the other studies using the decomposition approach^(20–22)^. A study by Cengiz *et al.* which evaluated changes in diet quality in the USA found that diet improvements in the USA were driven entirely by supply-side factors, while consumer switches worsened diet quality^(23)^. Spiteri and Soler found that the impact of consumer switching was different across categories, while reformulation consistently improved the nutrient density of products^(22)^. Contrary to the case of soft drink reformulation studied here, other studies using the decomposition approach focused on reformulation in the absence of a tax. This may explain why they found that demand-driven changes were limited: consumers were not incentivised to change their behaviour, and reformulation was likely less prominently advertised.

### Differences across households

Although the average sugar density of purchases decreased for both food and drinks, there were important differences across households. The analysis at the household level showed changes in the sugar density of purchases to be highly correlated with consumer switches (correlation above 80 %). Some demographic characteristics partly explained the types of switches made. Socio-economic group was associated with the size of the switching effect for all drinks. We found that households in lower socio-economic groups were more likely to decrease the sugar density of the products they purchased compared with households in higher socio-economic groups. However, this effect was not observed for switches in soft drinks, suggesting a switch to non-taxed products for households in lower socio-economic groups. A study evaluating the effect of the soft drinks tax in South Africa found similarly that the switching effect was stronger in the middle than in the higher socio-economic group^(24)^. This effect could be a result of early price increases in anticipation, or soon after the implementation, of the SDIL. The SDIL was responsible for price increases for taxed drinks of approximately 30 % (an increase of 7·5 pence/100 ml)^(25)^. Taxes on sugar-sweetened beverages had different effects across socio-economic groups in other countries: in Mexico, low-income groups purchased fewer drinks after the implementation of an SSB tax, but the reverse was observed in Chile^(26)^. In both of the above countries the cultural context around soft drink consumption may be different from that of the UK. Other studies evaluating the impact of the announcement and early implementation of the SDIL found different results from this study. One study using household data found a small increase in the quantities of sugar purchased between 2014 and March 2018 (i.e. before the implementation of the SDIL)^(4)^.

### The role of baseline household preference for sugar

For the three categories in the household-level analysis, a higher sugar density of purchases at baseline, used as a measure of the sugar preference of the household, was associated with a switching effect towards products with less sugar. In the literature, the opposite relationship was observed by which a higher preference for a nutrient was linked to lower odds of reducing one’s consumption. A study on the effects of awareness about the Mexican tax on soft drinks found that people with a higher liking for soft drinks were less likely to self-report a decrease in their consumption^(27)^. Similarly, UK individuals disliking soft drinks were more likely to perceive the SDIL as an effective policy^(28)^. The effect observed in this study could result from the choice options available to households. Households purchasing products with a higher sugar density at baseline had the possibility to switch to products with less sugar as these come onto the market.

Nonetheless, household characteristics and household habits and preferences can only partially explain the different behaviours observed across households, as also observed in a study on the acceptability of suggested food switches to products with lower salt, which found that the acceptability of food switches did not differ by sex, age, ethnic group, BMI, education or income^(29)^. This meant that the switching effect was mostly explained by unobserved variables. Furthermore, there was no correlation between the switching effect of drinks and of breakfast cereals. This means that it may not be effective to design education policies for population groups defined by their demographic characteristics, as this study failed to identify ideal target groups. Rather, switches between products may be driven by concerns for health. A study on food switches aiming at lowering salt purchases found that participants who considered health as important while choosing foods, or declared using food labels, had a larger reduction in salt purchased than those who did not consider health nor use food labels while choosing foods^(30)^.

### A consistent reformulation effect

Contrary to the high variability of the switching effect, denoting high variability between households’ behaviours, the reformulation effects had lower variability: the change in sugar density from reformulation was small and relatively homogeneous across households. Demographic characteristics were generally not associated with the reformulation effect, which means that reformulation had a similarly beneficial effect across all households. A study on total sugar purchases by UK households found no change in disparities (across social classes) in sugar purchases between 2014 and 2017^(10)^. During the study period, only the voluntary sugar reformulation programme was in place, the SDIL had been announced, which triggered action by manufacturers. The PHE evaluation of the first 3 years of the SDIL showed that different socio-economic groups reduced their sugar purchased from drinks in roughly equal quantities^(7)^. This homogeneous effect of reformulation across socio-economic groups indicates that reformulation is an equitable policy that is not expected to widen existing disparities. Using purchases from a large number of supermarkets in the UK, a study evaluated the role of reformulation and change in consumer behaviour on total population purchases between 2014 and 2020. Overall, it found that 20 % of the volume sold were from reformulated products^(31)^. It was estimated that 83 % of the sugar reduction observed in product sales came from the reformulation of products, while only 17 % came from changes in consumer behaviour (i.e. consumers switching to other brands)^(31)^. In comparison, we found that, on average across households, 30 % of the decrease in sugar density of drinks purchased came from changes in consumer behaviour and 38 % came from reformulation.

### Strengths and limitations

The main strength of this study is that it combined population-level and household-level analyses to investigate heterogeneity across households. To our knowledge, this is the first study that applies a decomposition approach at a disaggregated level and assesses heterogeneity in a population. Also, the decomposition approach was applied to disaggregated products, meaning that the reformulation effect measured relates to the actual reformulation of individual products.

Nonetheless, this study uses non-probabilistic and probabilistic methods that do not allow causal inference. This means that we do not know if the changes observed in purchasing patterns and their impact on sugar quantity purchased are caused by the reformulation of products or the implementation of the SDIL. In addition, we do not know if the reformulation observed was an effect of the different policies (i.e. the sugar reduction programme and the SDIL), or if it would have happened in the absence of them.

We also acknowledge some limitations regarding the use of household-level data. First, the household-level analysis does not capture purchases of food consumed out-of-home. Second, the aggregation of purchases at the level of a household prevents the study of individual behaviours, including differences between adults and children. Finally, the household-level decomposition does not cover all food categories, which means that we could not assess if compensation across categories took place, although we are reassured that the population-level analysis showed a decrease in the sugar density of all products purchased.

### Conclusions

The small reduction in the sugar density of foods purchased in the UK between 2015 and 2018 was generated by supply-side drivers only (reformulation and product renewal), whereas the larger reduction in the sugar density of drinks purchased was generated by both changes in supply and demand. Smaller reductions are seen in food categories compared with drinks as reducing sugar can be more difficult due to the functional role it plays in, for example, chocolate and sweet confectionary. It appears that the taxation of sugar-sweetened beverages created at the same time a stronger incentive for manufacturers to reformulate their products and pushed consumers to change their purchasing habits towards lower sugar products.

Switches to lower sugar products happened for products targeted by the SDIL, but not in the absence of such incentive. Switches between products were heterogeneous across households, but this heterogeneity was not associated with observable demographic characteristics of households.

The reformulation that happened in both foods and drinks had a homogenous effect across households of different socio-economic status, which makes reformulation consistent with health equity goals.

## Supporting information

Gressier et al. supplementary material Gressier et al. supplementary material

## Supplementary material

For supplementary material/s referred to in this article, please visit https://doi.org/10.1017/S0007114524001806
