# Supplementary material for: Supply- and demand-side drivers of the change in the sugar density of food purchased between 2015 and 2018 in Great Britain
Source: Br J Nutr. 2024 Oct 30;133(6):725–36. doi: 10.1017/S0007114524001806 (PMC12169944; doi:10.1017/S0007114524001806)
Supplement: Gressier et al. supplementary material [file S0007114524001806sup001.docx]

# On-line Supplementary Material

Investigating supply- and demand-side drivers of the change in the sugar density of food purchased between 2015 and 2018 in Great Britain

Authors: Mathilde Gressier, Gary Frost, Zoe Hill, Danying Li, Jack Olney, Elisa Pineda, Victoria Targett, Michelle Young, Franco Sassi

Correspondence to: [f.sassi@imperial.ac.uk](mailto:f.sassi@imperial.ac.uk)

# Supplementary figures

Supplementary Figure 1: Distribution (boxplots) of the change in sugar density (g sugar per 100ml) of all drinks, purchased by households between 2015 and 2018, and its decomposition into reformulation, switching and product renewal effects


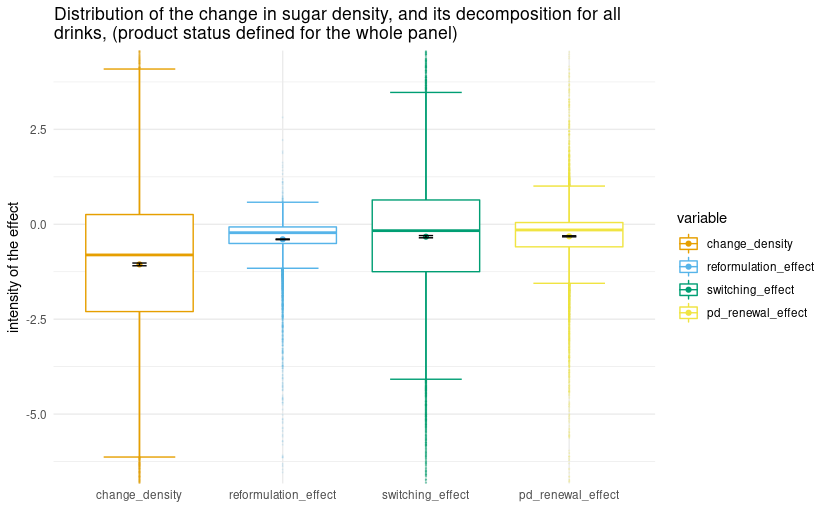


Intensity of the effect (g/100mL)

Note: Boxes show the 1^st^ quarter, median and 3^rd^ quarter, the dots outside of the whiskers show extreme points; the black point and error bar show mean and standard error

Data source: Kantar panel, household level. The decomposition method was applied for each household

Supplementary Figure 2: Distribution of the change in sugar density (g sugar per 100ml) of soft drinks, purchased by households between 2015 and 2018, and its decomposition into reformulation, switching and product renewal effects


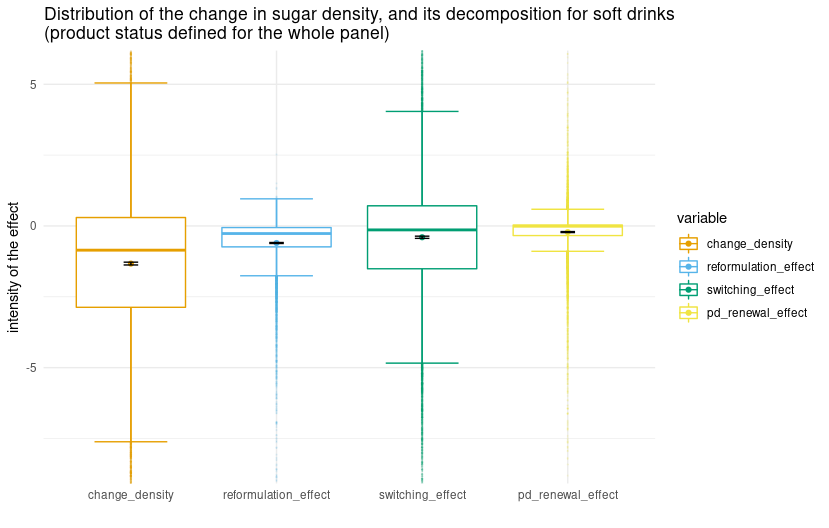


Intensity of the effect (g/100mL)

Note: Boxes show the 1^st^ quarter, median and 3^rd^ quarter, the dots outside of the whiskers show extreme points; the black point and error bar show mean and standard error

Data source: Kantar panel, household level. The decomposition method was applied for each household

Supplementary Figure 3: Distribution of the change in sugar density (g sugar per 100g) of breakfast cereals, purchased by households between 2015 and 2018, and its decomposition into reformulation, switching and product renewal effects


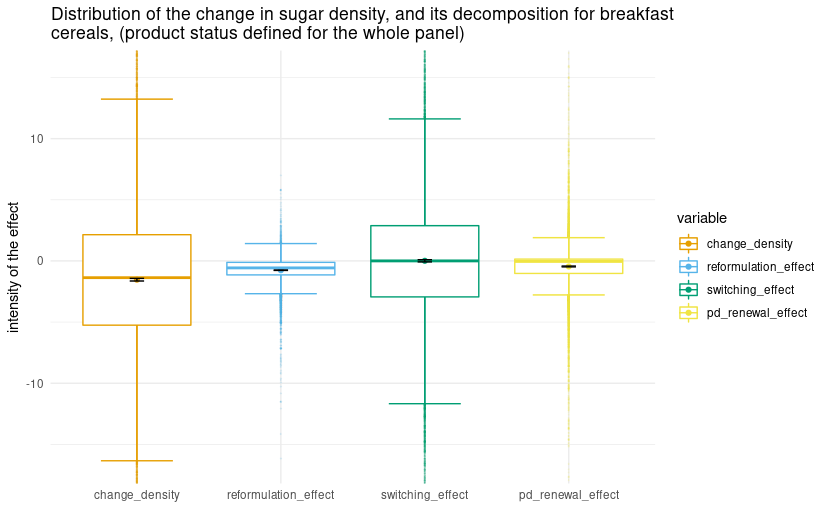


Intensity of the effect (g/100g)

Note: Boxes show the 1^st^ quarter, median and 3^rd^ quarter, the dots outside of the whiskers show extreme points; the black point and error bar show mean and standard error

Data source: Kantar panel, household level. The decomposition method was applied for each household

Supplementary Figure 4: Predicted effect of household life-stage and the sugar density of breakfast cereal purchases on the switching effect for the breakfast cereal category.


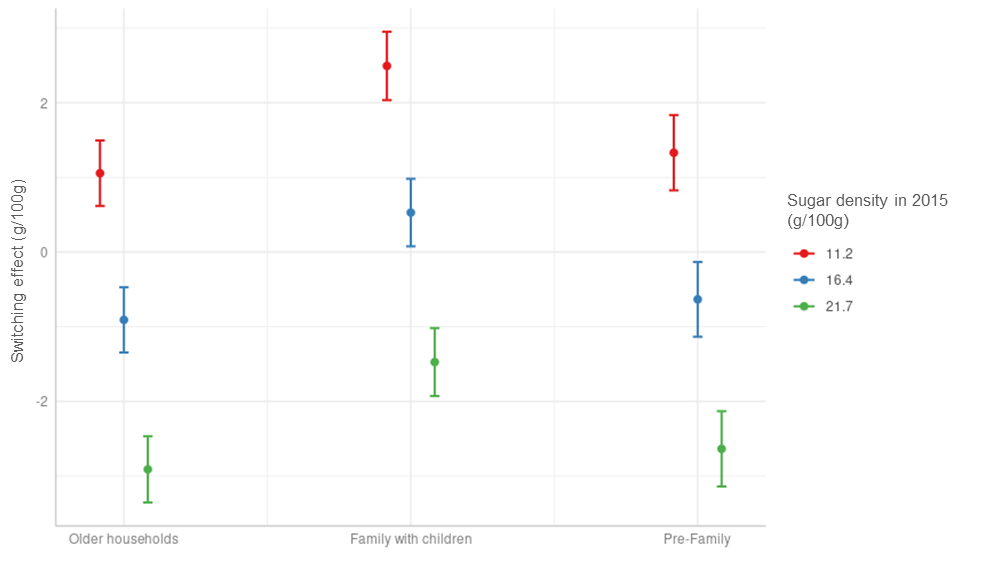


*Notes*: Results from equation 2. The effects are predicted at the 25^th^, 50^th^ and 75^th^ centile of the distribution of the baseline sugar density.

Data source: Kantar panel, household level.

Supplementary Figure 5: Predicted mean effect of social grade and the baseline volume of soft drinks purchased by adult equivalent (AE) in the household on the purchase of sugar from soft drinks


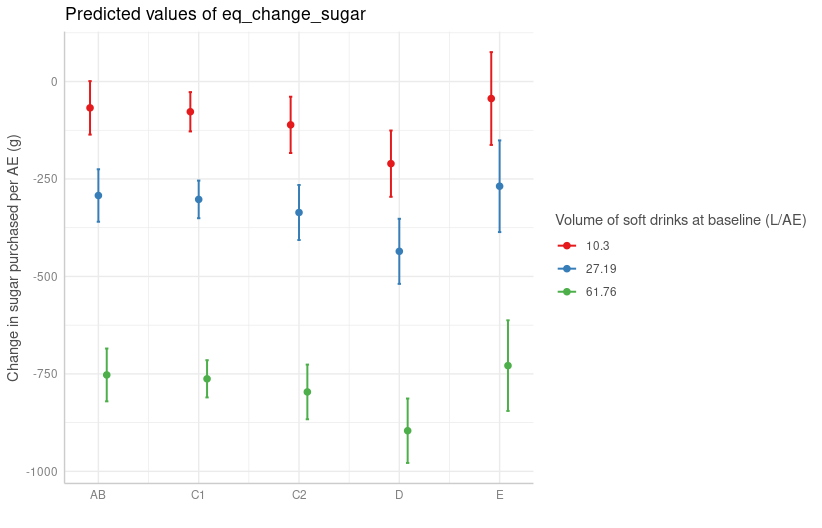


*Notes*: Results from equation 3. The effects were modelled at the 25^th^, 50^th^, and 75^th^ centile of baseline volume of soft drinks per adult equivalent purchased. Bars show the 95% confidence intervals.

Data source: Kantar panel, household level.

Supplementary Figure 6: Predicted mean effect of the social grade of the household on the purchase of sugar from breakfast cereals


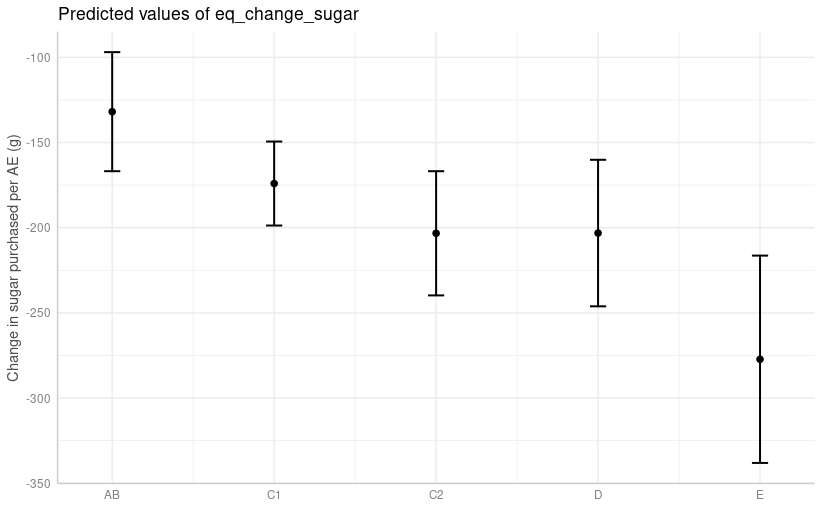


*Note*: Results from equation 3. Bars show the 95% confidence interval.

Data source: Kantar panel, household level.

# Supplementary tables

Supplementary Table 1: Descriptive statistics of the decomposition of the changes in sugar density of household purchases between 2015 and 2018, into the effects of reformulation, product renewal and switches (in g/100g or g/100mL).

|  |  | All drinks (n=18512) | |  | Soft drinks (n=18343) | |  | Breakfast cereals (n=18533) | |
| --- | --- | --- | --- | --- | --- | --- | --- | --- | --- |
|  |  | value | SE |  | value | SE |  | value | SE |
| **change in sugar density** | Mean | -1.06 | 0.02 |  | -1.33 | 0.03 |  | -1.53 | 0.05 |
|  | Q25 | -2.3 |  |  | -2.87 |  |  | -5.25 |  |
|  | Median | -0.81 |  |  | -0.85 |  |  | -1.37 |  |
|  | Q75 | 0.26 |  |  | 0.3 |  |  | 2.14 |  |
| **reformulation effect** | Mean | -0.4 | 0 |  | -0.6 | 0.01 |  | -0.76 | 0.01 |
|  | Q25 | -0.51 |  |  | -0.74 |  |  | -1.14 |  |
|  | Median | -0.22 |  |  | -0.27 |  |  | -0.57 |  |
|  | Q75 | -0.07 |  |  | -0.06 |  |  | -0.12 |  |
| **product renewal effect** | Mean | -0.32 | 0.01 |  | -0.22 | 0.01 |  | -0.45 | 0.02 |
|  | Q25 | -0.59 |  |  | -0.34 |  |  | -1.02 |  |
|  | Median | -0.15 |  |  | -0.01 |  |  | -0.04 |  |
|  | Q75 | 0.05 |  |  | 0.03 |  |  | 0.15 |  |
| **switching effect** | Mean | -0.33 | 0.01 |  | -0.4 | 0.02 |  | 0.01 | 0.05 |
|  | Q25 | -1.25 |  |  | -1.51 |  |  | -2.94 |  |
|  | Median | -0.17 |  |  | -0.14 |  |  | 0 |  |
|  | Q75 | 0.64 |  |  | 0.71 |  |  | 2.88 |  |

Results of the decomposition algorithm, applied for each household in the panel. SE: standard error (of the mean). Number in parenthesis show the number of households used in each analysis (households having recorded purchases of these products).

Data source: Kantar panel, household level.

Supplementary Table 2: Regression results for all drinks: prediction of decomposition effects (in g/100L) with household characteristics and preferences (equation 2)

|  | reformulation effect | | | switching effect | | | product renewal effect | | |
| --- | --- | --- | --- | --- | --- | --- | --- | --- | --- |
| term | estimate | SE | p.value | estimate | SE | p.value | estimate | SE | p.value |
| (Intercept) | 0.09 | 0.03 | **0.008** | 1.30 | 0.11 | **0.000** | 0.28 | 0.05 | **0.000** |
| Volume of purchases in 2015 | -8.5E-05 | 5E-05 | 0.082 | -9.3E-04 | 2E-04 | **0.000** | -3.8E-04 | 7E-05 | **0.000** |
| Mean sugar density of purchases in 2015 | -0.10 | 0.00 | **0.000** | -0.27 | 0.00 | **0.000** | -0.10 | 0.00 | **0.000** |
| N household members | 0.00 | 0.00 | 0.361 | -0.03 | 0.02 | **0.025** | -0.02 | 0.01 | **0.001** |
| Household.Income£10,000 - £19,999 pa | -0.01 | 0.02 | 0.615 | -0.08 | 0.06 | 0.192 | 0.02 | 0.03 | 0.612 |
| Household.Income£20,000 - £29,999 pa | 0.01 | 0.02 | 0.801 | -0.10 | 0.07 | 0.139 | 0.04 | 0.03 | 0.242 |
| Household.Income£30,000 - £39,999 pa | 0.00 | 0.02 | 0.951 | -0.15 | 0.07 | **0.032** | 0.02 | 0.03 | 0.649 |
| Household.Income£40,000 - £49,999 pa | 0.03 | 0.02 | 0.258 | -0.24 | 0.08 | **0.002** | 0.00 | 0.04 | 0.989 |
| Household.Income£50,000 - £59,999 pa | 0.01 | 0.03 | 0.673 | -0.26 | 0.08 | **0.002** | 0.00 | 0.04 | 0.981 |
| Household.Income£60,000 - £69,999 pa | 0.00 | 0.03 | 0.947 | -0.11 | 0.10 | 0.256 | 0.03 | 0.05 | 0.543 |
| Household.Income£70,000 + | 0.03 | 0.03 | 0.316 | -0.16 | 0.09 | 0.080 | 0.04 | 0.04 | 0.410 |
| Household.IncomeDid not want to answer | 0.00 | 0.02 | 0.892 | -0.09 | 0.07 | 0.173 | 0.04 | 0.03 | 0.234 |
| Social.GradeC1 | -0.01 | 0.01 | 0.255 | -0.04 | 0.04 | 0.268 | -0.01 | 0.02 | 0.549 |
| Social.GradeC2 | -0.01 | 0.01 | 0.322 | -0.14 | 0.05 | **0.002** | -0.05 | 0.02 | **0.018** |
| Social.GradeD | 0.00 | 0.02 | 0.839 | -0.18 | 0.05 | **0.000** | -0.04 | 0.02 | 0.079 |
| Social.GradeE | -0.01 | 0.02 | 0.775 | -0.22 | 0.07 | **0.001** | -0.01 | 0.03 | 0.780 |
| Urban...Rural | 0.02 | 0.05 | 0.616 | 0.15 | 0.15 | 0.339 | 0.01 | 0.07 | 0.899 |
| Urban...RuralRural | 0.03 | 0.02 | 0.261 | 0.21 | 0.08 | **0.005** | 0.11 | 0.04 | **0.004** |
| Urban...RuralSemi-rural | 0.04 | 0.02 | **0.027** | 0.06 | 0.06 | 0.279 | 0.07 | 0.03 | **0.011** |
| Urban...RuralSuburban | 0.01 | 0.02 | 0.403 | 0.03 | 0.05 | 0.550 | 0.05 | 0.02 | **0.018** |
| Urban...RuralUrban | 0.03 | 0.02 | 0.093 | -0.07 | 0.05 | 0.125 | 0.01 | 0.02 | 0.735 |
| Ethnicityother | -0.03 | 0.02 | 0.065 | 0.21 | 0.05 | **0.000** | 0.00 | 0.02 | 0.946 |
| Life_stageOlder households | -0.05 | 0.01 | **0.000** | -0.07 | 0.04 | 0.083 | -0.04 | 0.02 | **0.042** |
| Life_stagePre-Family | -0.03 | 0.02 | 0.164 | -0.05 | 0.06 | 0.376 | -0.03 | 0.03 | 0.335 |
| RegionEast | -0.01 | 0.02 | 0.734 | -0.06 | 0.07 | 0.378 | -0.02 | 0.03 | 0.609 |
| RegionMidlands | 0.01 | 0.02 | 0.673 | -0.06 | 0.06 | 0.352 | -0.09 | 0.03 | **0.002** |
| RegionNorth | 0.04 | 0.02 | **0.021** | -0.10 | 0.06 | 0.078 | -0.09 | 0.03 | **0.001** |
| RegionScotland | -0.04 | 0.02 | 0.068 | 0.04 | 0.07 | 0.542 | -0.04 | 0.03 | 0.211 |
| RegionSouth | 0.01 | 0.02 | 0.726 | 0.01 | 0.06 | 0.869 | -0.03 | 0.03 | 0.341 |
| RegionWales | 0.09 | 0.03 | **0.001** | -0.02 | 0.08 | 0.796 | -0.09 | 0.04 | **0.015** |
| R² | 0.23 | | | 0.15 | | | 0.11 | | |

Data source: Kantar panel, household level.

Supplementary Table 3: Regression results for soft drinks: prediction of decomposition effects (in g/100mL) with household characteristics and preferences (equation 2)

|  | reformulation effect | | | switching effect | | | product renewal effect | | |
| --- | --- | --- | --- | --- | --- | --- | --- | --- | --- |
| term | estimate | SE | p.value | estimate | SE | p.value | estimate | SE | p.value |
| (Intercept) | 0.17 | 0.05 | **0.002** | 1.86 | 0.13 | **0.000** | 0.36 | 0.06 | **0.000** |
| Volume of purchases in 2015 | -4.2E-05 | 1E-04 | 0.713 | -2.9E-03 | 3E-04 | **0.000** | -5.9E-04 | 1E-04 | **0.000** |
| Mean sugar density of purchases in 2015 | -0.17 | 0.00 | **0.000** | -0.31 | 0.00 | **0.000** | -0.10 | 0.00 | **0.000** |
| N household members | 0.02 | 0.01 | **0.025** | -0.10 | 0.02 | **0.000** | -0.01 | 0.01 | 0.105 |
| Household.Income£10,000 - £19,999 pa | 0.02 | 0.03 | 0.588 | -0.13 | 0.08 | 0.112 | 0.00 | 0.04 | 0.978 |
| Household.Income£20,000 - £29,999 pa | 0.03 | 0.04 | 0.434 | -0.18 | 0.09 | **0.040** | -0.01 | 0.04 | 0.838 |
| Household.Income£30,000 - £39,999 pa | 0.04 | 0.04 | 0.295 | -0.15 | 0.09 | 0.105 | -0.06 | 0.04 | 0.119 |
| Household.Income£40,000 - £49,999 pa | 0.05 | 0.04 | 0.249 | -0.31 | 0.10 | **0.001** | -0.02 | 0.04 | 0.674 |
| Household.Income£50,000 - £59,999 pa | 0.06 | 0.04 | 0.196 | -0.50 | 0.11 | **0.000** | 0.03 | 0.05 | 0.563 |
| Household.Income£60,000 - £69,999 pa | -0.01 | 0.05 | 0.912 | -0.30 | 0.13 | **0.017** | -0.04 | 0.06 | 0.427 |
| Household.Income£70,000 + | 0.03 | 0.05 | 0.552 | -0.23 | 0.12 | **0.050** | -0.07 | 0.05 | 0.202 |
| Household.IncomeDid not want to answer | 0.02 | 0.04 | 0.538 | -0.09 | 0.09 | 0.291 | 0.00 | 0.04 | 0.970 |
| Social.GradeC1 | 0.03 | 0.02 | 0.114 | -0.04 | 0.05 | 0.436 | -0.01 | 0.02 | 0.709 |
| Social.GradeC2 | 0.06 | 0.02 | **0.021** | -0.09 | 0.06 | 0.144 | -0.01 | 0.03 | 0.846 |
| Social.GradeD | 0.08 | 0.03 | **0.002** | 0.00 | 0.07 | 0.949 | -0.03 | 0.03 | 0.274 |
| Social.GradeE | 0.10 | 0.03 | **0.003** | 0.02 | 0.08 | 0.828 | -0.02 | 0.04 | 0.565 |
| Urban...Rural | 0.03 | 0.08 | 0.724 | -0.18 | 0.19 | 0.360 | -0.01 | 0.09 | 0.884 |
| Urban...RuralRural | 0.02 | 0.04 | 0.627 | 0.09 | 0.10 | 0.350 | 0.07 | 0.04 | 0.102 |
| Urban...RuralSemi-rural | 0.03 | 0.03 | 0.280 | -0.14 | 0.08 | 0.069 | 0.03 | 0.03 | 0.348 |
| Urban...RuralSuburban | 0.01 | 0.02 | 0.688 | -0.12 | 0.06 | **0.049** | 0.00 | 0.03 | 0.929 |
| Urban...RuralUrban | 0.05 | 0.03 | 0.068 | -0.19 | 0.06 | **0.002** | 0.01 | 0.03 | 0.751 |
| Ethnicityother | -0.01 | 0.03 | 0.779 | 0.32 | 0.07 | **0.000** | 0.07 | 0.03 | **0.016** |
| Life_stageOlder households | -0.09 | 0.02 | **0.000** | -0.04 | 0.05 | 0.473 | 0.01 | 0.02 | 0.773 |
| Life_stagePre-Family | -0.06 | 0.03 | 0.066 | -0.05 | 0.07 | 0.480 | 0.00 | 0.03 | 0.923 |
| RegionEast | -0.09 | 0.03 | **0.008** | -0.02 | 0.09 | 0.798 | -0.04 | 0.04 | 0.245 |
| RegionMidlands | -0.08 | 0.03 | **0.010** | -0.12 | 0.08 | 0.127 | -0.07 | 0.04 | 0.060 |
| RegionNorth | -0.03 | 0.03 | 0.276 | -0.22 | 0.07 | **0.003** | -0.07 | 0.03 | **0.042** |
| RegionScotland | -0.15 | 0.04 | **0.000** | -0.02 | 0.09 | 0.857 | -0.07 | 0.04 | 0.073 |
| RegionSouth | -0.09 | 0.03 | **0.005** | 0.01 | 0.08 | 0.938 | -0.05 | 0.03 | 0.120 |
| RegionWales | 0.03 | 0.04 | 0.415 | -0.05 | 0.11 | 0.653 | -0.15 | 0.05 | **0.001** |
| R² | 0.33 | | | 0.20 | | | 0.11 | | |

Data source: Kantar panel, household level.

Supplementary Table 4: Regression results for breakfast cereals: prediction of decomposition effects (in g/100g) with household characteristics and preferences (equation 2)

|  | reformulation effect | | | switching effect | | | product renewal effect | | |
| --- | --- | --- | --- | --- | --- | --- | --- | --- | --- |
| term | estimate | SE | p.value | estimate | SE | p.value | estimate | SE | p.value |
| (Intercept) | 0.09 | 0.06 | 0.108 | 7.16 | 0.32 | **0.000** | 1.11 | 0.14 | **0.000** |
| eq_volume_2015 | -7.0E-03 | 9E-04 | **0.000** | -2.8E-02 | 5E-03 | **0.000** | -6.4E-03 | 2E-03 | **0.002** |
| densityHH15 | -0.05 | 0.00 | **0.000** | -0.38 | 0.01 | **0.000** | -0.08 | 0.00 | **0.000** |
| N_people | -0.02 | 0.01 | **0.034** | 0.07 | 0.05 | 0.148 | 0.02 | 0.02 | 0.410 |
| Household.Income£10,000 - £19,999 pa | 0.03 | 0.04 | 0.332 | -0.36 | 0.19 | 0.062 | -0.10 | 0.08 | 0.221 |
| Household.Income£20,000 - £29,999 pa | 0.06 | 0.04 | 0.096 | -0.34 | 0.20 | 0.087 | -0.11 | 0.09 | 0.204 |
| Household.Income£30,000 - £39,999 pa | 0.06 | 0.04 | 0.135 | -0.27 | 0.21 | 0.206 | -0.17 | 0.09 | 0.057 |
| Household.Income£40,000 - £49,999 pa | 0.05 | 0.04 | 0.242 | -0.36 | 0.22 | 0.110 | -0.16 | 0.10 | 0.090 |
| Household.Income£50,000 - £59,999 pa | 0.03 | 0.05 | 0.468 | -0.24 | 0.25 | 0.330 | -0.08 | 0.11 | 0.448 |
| Household.Income£60,000 - £69,999 pa | 0.12 | 0.05 | **0.031** | -0.45 | 0.29 | 0.122 | -0.09 | 0.13 | 0.478 |
| Household.Income£70,000 + | 0.11 | 0.05 | **0.026** | -0.20 | 0.27 | 0.460 | -0.19 | 0.12 | 0.098 |
| Household.IncomeDid not want to answer | 0.04 | 0.04 | 0.332 | -0.43 | 0.21 | **0.039** | -0.15 | 0.09 | 0.083 |
| Social.GradeC1 | 0.00 | 0.02 | 0.833 | 0.15 | 0.11 | 0.192 | 0.02 | 0.05 | 0.646 |
| Social.GradeC2 | 0.03 | 0.02 | 0.194 | 0.15 | 0.14 | 0.282 | 0.10 | 0.06 | 0.097 |
| Social.GradeD | 0.02 | 0.03 | 0.405 | 0.27 | 0.15 | 0.074 | 0.09 | 0.07 | 0.154 |
| Social.GradeE | 0.01 | 0.04 | 0.676 | 0.16 | 0.19 | 0.409 | 0.00 | 0.08 | 0.989 |
| Urban...Rural | 0.11 | 0.08 | 0.180 | -0.02 | 0.45 | 0.965 | -0.35 | 0.19 | 0.067 |
| Urban...RuralRural | 0.01 | 0.04 | 0.833 | -0.67 | 0.23 | **0.003** | -0.02 | 0.10 | 0.865 |
| Urban...RuralSemi-rural | 0.01 | 0.03 | 0.809 | -0.23 | 0.18 | 0.201 | -0.10 | 0.08 | 0.197 |
| Urban...RuralSuburban | 0.02 | 0.03 | 0.437 | -0.27 | 0.14 | 0.058 | -0.09 | 0.06 | 0.140 |
| Urban...RuralUrban | 0.03 | 0.03 | 0.188 | -0.02 | 0.15 | 0.868 | -0.06 | 0.06 | 0.326 |
| Ethnicityother | 0.01 | 0.03 | 0.752 | 0.48 | 0.16 | **0.002** | 0.18 | 0.07 | **0.007** |
| Life_stageOlder households | 0.00 | 0.02 | 0.874 | -1.44 | 0.12 | **0.000** | -0.07 | 0.05 | 0.208 |
| Life_stagePre-Family | 0.01 | 0.03 | 0.635 | -1.16 | 0.17 | **0.000** | -0.12 | 0.07 | 0.114 |
| RegionEast | -0.03 | 0.04 | 0.435 | 0.31 | 0.20 | 0.120 | -0.07 | 0.08 | 0.433 |
| RegionMidlands | 0.00 | 0.03 | 0.941 | 0.42 | 0.18 | **0.023** | -0.01 | 0.08 | 0.871 |
| RegionNorth | 0.02 | 0.03 | 0.470 | 0.43 | 0.17 | **0.014** | -0.01 | 0.07 | 0.926 |
| RegionScotland | -0.15 | 0.04 | **0.000** | 0.69 | 0.21 | **0.001** | 0.11 | 0.09 | 0.224 |
| RegionSouth | -0.02 | 0.03 | 0.589 | 0.31 | 0.18 | 0.075 | -0.08 | 0.08 | 0.282 |
| RegionWales | -0.10 | 0.04 | **0.027** | 0.61 | 0.25 | **0.012** | 0.02 | 0.10 | 0.877 |
| R² | 0.13 | | | 0.20 | | | 0.06 | | |

Data source: Kantar panel, household level.

Supplementary Table 5: Regression of the change in sugar quantity purchased (in g/year) per adult-equivalent on household characteristics and decomposition results (equation 3)

|  | **All drinks** | | | **Soft drinks** | | | **Breakfast cereals** | | |
| --- | --- | --- | --- | --- | --- | --- | --- | --- | --- |
|  | estimate | SE | p.value | estimate | SE | p.value | estimate | SE | p.value |
| (Intercept) | 1905.4 | 246.2 | **0.000** | 778.4 | 194.3 | **0.000** | 816.2 | 102.9 | **0.000** |
| reformulation effect | 337.4 | 32.5 | **0.000** | 73.8 | 16.2 | **0.000** | 77.3 | 7.8 | **0.000** |
| switching effect | 504.8 | 10.2 | **0.000** | 204.4 | 6.5 | **0.000** | 61.5 | 1.4 | **0.000** |
| product renewal effect | 522.9 | 21.4 | **0.000** | 128.8 | 14.4 | **0.000** | 60.1 | 3.3 | **0.000** |
| Mean sugar density of purchases in 2015 | -148.9 | 8.3 | **0.000** | -126.7 | 5.9 | **0.000** | -7.5 | 1.3 | **0.000** |
| Volume of purchases in 2015 | -13.2 | 0.2 | **0.000** | -13.3 | 0.2 | **0.000** | -58.5 | 0.9 | **0.000** |
| N household members | -186.1 | 20.8 | **0.000** | -85.4 | 16.5 | **0.000** | -78.4 | 8.7 | **0.000** |
| Household Income £10,000 - £19,999 pa | 66.2 | 88.3 | 0.453 | 120.6 | 70.7 | 0.088 | 57.0 | 36.9 | 0.122 |
| Household Income £20,000 - £29,999 pa | 201.9 | 92.5 | **0.029** | 223.0 | 74.1 | **0.003** | -4.8 | 38.6 | 0.901 |
| Household Income £30,000 - £39,999 pa | 38.2 | 97.2 | 0.694 | 122.2 | 77.8 | 0.116 | 22.2 | 40.5 | 0.584 |
| Household Income £40,000 - £49,999 pa | 96.5 | 103.5 | 0.351 | 130.9 | 82.7 | 0.114 | 13.7 | 43.1 | 0.750 |
| Household Income £50,000 - £59,999 pa | -5.2 | 115.1 | 0.964 | 158.5 | 91.9 | 0.084 | -38.0 | 47.8 | 0.427 |
| Household Income £60,000 - 69,999 pa | 92.6 | 135.5 | 0.494 | 94.7 | 107.9 | 0.381 | 23.2 | 56.3 | 0.680 |
| Household Income >£70,000 | 40.1 | 125.2 | 0.748 | 125.3 | 100.0 | 0.210 | 59.2 | 52.1 | 0.255 |
| Household Income Did not want to answer | 64.0 | 94.8 | 0.500 | 93.3 | 75.9 | 0.219 | 31.7 | 39.6 | 0.423 |
| Socioeconomic group C1 | 0.8 | 52.3 | 0.987 | -10.0 | 41.6 | 0.811 | -42.2 | 21.7 | 0.052 |
| Socioeconomic group C2 | 14.6 | 63.2 | 0.817 | -43.6 | 50.3 | 0.386 | -71.4 | 26.2 | **0.007** |
| Socioeconomic group D | -125.5 | 71.1 | 0.077 | -143.1 | 56.6 | **0.012** | -71.2 | 29.5 | **0.016** |
| Socioeconomic group E | 90.9 | 89.8 | 0.311 | 23.9 | 71.6 | 0.739 | -145.3 | 37.3 | **0.000** |
| City | 9.6 | 209.2 | 0.963 | 15.9 | 165.9 | 0.924 | -22.1 | 86.5 | 0.799 |
| Rural | -48.6 | 219.0 | 0.824 | -20.4 | 173.7 | 0.906 | 7.4 | 90.6 | 0.935 |
| Semi-rural | 133.7 | 208.9 | 0.522 | 135.0 | 165.7 | 0.415 | 65.5 | 86.4 | 0.449 |
| Suburban | 49.0 | 203.8 | 0.810 | 57.4 | 161.6 | 0.722 | 32.1 | 84.3 | 0.703 |
| Urban | 57.8 | 204.4 | 0.777 | 54.7 | 162.1 | 0.736 | 16.6 | 84.5 | 0.845 |
| Ethnicity (other than white) | 17.6 | 72.2 | 0.807 | 68.2 | 57.5 | 0.236 | 3.7 | 29.9 | 0.903 |
| Life stage = Older households | -319.8 | 55.8 | **0.000** | -88.0 | 44.3 | **0.047** | -193.8 | 23.3 | **0.000** |
| Life stage = Pre-Family | -188.3 | 79.2 | **0.018** | -60.0 | 63.0 | 0.341 | -112.0 | 33.0 | **0.001** |
| Region East | 168.8 | 91.5 | 0.065 | 136.4 | 73.1 | 0.062 | 104.3 | 38.0 | **0.006** |
| Region Midlands | 73.9 | 84.6 | 0.382 | 109.4 | 67.5 | 0.105 | 97.2 | 35.2 | **0.006** |
| Region North | 150.4 | 79.8 | 0.060 | 139.4 | 63.7 | **0.029** | 86.7 | 33.2 | **0.009** |
| Region Scotland | 110.5 | 95.7 | 0.248 | 157.2 | 76.5 | **0.040** | 28.7 | 39.9 | 0.472 |
| Region South | 168.4 | 81.2 | **0.038** | 101.0 | 64.9 | 0.119 | 111.8 | 33.8 | **0.001** |
| Region Wales | 222.3 | 112.9 | **0.049** | 194.3 | 90.1 | **0.031** | 51.9 | 47.0 | 0.269 |
| R² | 0.34 | | | 0.27 | | | 0.31 | | |

Data source: Kantar panel, household level.
